# Smoking exposure alters splicing of the nicotinic acetylcholine receptor subunit *CHRNA5*

**DOI:** 10.1186/s40246-026-01004-y

**Published:** 2026-06-16

**Authors:** Maxwell H. Hogshead, Atuahene Adu-Gyamfi, Brenen W. Papenberg, Wusheng Yan, Chia-Han Lee, Oscar Florez-Vargas, Ludmila Prokunina-Olsson

**Affiliations:** 1 Laboratory of Translational Genomics, Division of Cancer Epidemiology and Genetics, National Cancer Institute, National Institutes of Health, 9615 Medical Center Drive, Rockville, MD 20850 USA; 2 Frederick National Laboratory for Cancer Research, Bethesda, MD USA

**Keywords:** Nicotinic acetylcholine receptor (nAChR), Cigarette smoking, Exposomics, Cancer risk, Alternative splicing, Gene expression, Cloning, mRNA

## Abstract

**Background:**

Cigarette smoking is the major preventable cancer risk factor. The missense variant rs16969968 (G > A, D398N) in exon 5 of *CHRNA5* on chromosome 15q25.1 alters activity of the nicotinic acetylcholine receptor (α5-nAChR) and is associated with increased smoking intensity and cancer risk. We hypothesized that smoking exposure might affect alternative splicing of *CHRNA5*, which is mainly limited to exon 5, and thereby modulate genetic associations reported for rs16969968.

**Results:**

In human tissues and cell lines, we detected five main alternative *CHRNA5* isoforms, each lacking 130–786 bp of exon 5, including the region with rs16969968. In tumors from The Cancer Genome Atlas (TCGA), only 52.4% of all exon 5 splice junctions corresponded to the full-length isoform. We modeled cigarette smoking exposure by treating cell lines representing smoking-related cancers - A549 (lung) and UMUC3 (bladder) - with cigarette smoke concentrate (CSC). Splicing of *CHRNA5*-exon 5 was evaluated in the context of a transiently expressed Exontrap-*CHRNA5*-exon5 minigene using long-read targeted cDNA sequencing and RT-qPCR. Long-term, but not short-term, smoking exposure significantly altered the relative abundance of several *CHRNA5* splice isoforms, but independently of rs16969968 alleles.

**Conclusions:**

Smoking exposure can modulate *CHRNA5* splicing and, consequently, the composition and function of α5-nAChR, the receptor regulating the response to smoking. Thus, smoking-induced alterations of *CHRNA5-*exon 5 splicing can influence nicotine dependence and cancer risk, acting both independently of and complementary to the genetic risk conferred by the rs16969968-A variant.

**Supplementary Information:**

The online version contains supplementary material available at 10.1186/s40246-026-01004-y.

## Background

Cigarette smoking accounted for an estimated 101,300 lung cancer deaths in the United States in 2024 alone [1, 2]. In addition to lung cancer, smoking substantially elevates the risk of at least eleven other malignancies [1, 3], including bladder cancer, of which 50–60% are attributable to tobacco use [3].

Susceptibility to smoking-related outcomes, such as nicotine dependence and cancer risk, is significantly shaped by genetic factors [4–8]. One prominent example is the missense single-nucleotide polymorphism (SNP) rs16969968 (G > A, D398N) in exon 5 of *CHRNA5* at chromosome 15q25.1 [7–9]. *CHRNA5* encodes the α5 subunit of the neuronal nicotinic acetylcholine receptor (nAChR), a pentameric ligand-gated ion channel assembled from the α (α2–α10) and β (β2–β4) subunits [10]. The α5 subunit is incorporated into various nAChR assemblies [10], such as (α3β2)₂α5, (α3β4)₂α5, and (α4β2)₂α5, here collectively referred to as α5-nAChRs. Although it is considered an accessory subunit and not directly involved in ligand binding, the α5 subunit enhances cation permeability and modulates nicotine-induced signaling through α5-nAChRs [10]. The rs16969968-A (N398) risk allele reduces α5-nAChR sensitivity to nicotine [6, 11], leading to increased smoking intensity in carriers to achieve receptor activation levels comparable to those of noncarriers [12], thereby elevating their carcinogenic exposure and cancer risk [7, 8].

Here, we analyzed splicing profiles of five main alternative isoforms of *CHRNA5* detected in human tissues and cell lines, each isoform lacking 130–786 bp of exon 5, including the region encompassing rs16969968. Four of these isoforms have been partially annotated in a previous report [13]. Frameshifts and premature stop codons introduced by the alternative splicing events truncate the CHRNA5 protein by 89–308 amino acids (aa).

We hypothesized that splicing of *CHRNA5*, which encodes a subunit of the nicotine-responsive receptor, might be affected by smoking, thereby altering its functional response. We experimentally investigated the impact of smoking exposure by treating cell lines representing smoking-related cancers - A549 (lung cancer) and UMUC3 (bladder cancer) - with cigarette smoke concentrate (CSC). Splicing patterns of *CHRNA5*-exon5 were explored in an endogenous context and in a transiently transfected Exontrap-*CHRNA5*-exon5 minigene. Long-term (> 1 month), but not short-term (48 h), continuous CSC exposure significantly altered *CHRNA5* exon 5 splicing. These findings support a model in which environmental factors, such as cigarette smoke constituents, regulate α5-nAChR signaling by modulating *CHRNA5*-exon 5 splicing. This regulation may affect α5-nAChR trafficking and turnover, membrane stability, cation permeability, and downstream signaling pathways relevant to the regulation of smoking behavior and cancer risk.

## Methods

### Cell lines

A549 (CCL-185), a lung adenocarcinoma cell line, UMUC3 (CRL-1749), a bladder transitional carcinoma cell line, and SH-SY5Y (CRL-2266), a neuroblastoma cell line, were purchased from ATCC (Manassas). All cells were maintained in the recommended media - F-12 K, EMEM, and a 1:1 mixture of EMEM and F12, respectively - supplemented with 10% fetal bovine serum and a 1% antibiotic‒antimycotic mixture (100x, Thermo Fisher Scientific). Cells were routinely assessed for Mycoplasma contamination using the MycoAlert Mycoplasma Detection Kit (Lonza) and, if maintained for more than 6 months after purchase, authenticated annually using the AmpFLSTR Identifiler Plus Kit (Thermo Fisher Scientific).

### Analysis of public genomic data

The human CHRNA5 protein was annotated based on information from UniProt https://www.uniprot.org/uniprotkb/P30532/entry. Protein topology was visualized with Protter v1.0 [14] (http://wlab.ethz.ch/protter/). Splicing analysis of *CHRNA5* in The Cancer Genome Atlas (TCGA, 33 tumor types from 11,348 individuals) and the Genotype-Tissue Expression project (GTEx v8, 19,081 normal tissue samples from 971 individuals) was performed based on precomputed splice junction counts retrieved from the Recount3 project: uniformly processed RNA-seq (https://rna.recount.bio/) [15]. Tumor/tissue types with ≥ 10 samples and ≥ 30 splice junctions across all *CHRNA5*-exon 5 isoforms per sample were selected, yielding 384 TCGA tumors of 13 types, while no GTEx samples met these inclusion criteria.

Germline genotypes of rs16969968 in cell lines were determined by examining whole-genome sequencing (WGS) BAM files in the Integrative Genomics Viewer (IGV, v.2.19.1). Long-read WGS datasets were from SRA NCBI BioProjects PRJNA1178369 for A549 and PRJNA1134701 for UMUC3 [16]. A short-read WGS dataset for SH-SY5Y was from the Cancer Cell Line Encyclopedia (CCLE) (https://app.terra.bio/#workspaces/broad-firecloud-ccle/CCLE-public). The genotypes were also validated by direct genotyping of DNA samples from these cell lines with a TaqMan assay (described in the Allelic Expression Imbalance section).

Germline genotypes of rs16969968 in TCGA bladder cancer dataset (BLCA-TCGA) were determined from WGS data of blood-derived DNA using BCFtools mpileup (v1.20) [17]. Only samples with ≥ 10 read counts at rs16969968 were retained (*n* = 357). Heterozygous individuals (rs16969968-A/G, *n* = 149) were selected for paired (DNA-RNA) analyses in tumors. Of those, samples from 101 individuals with ≥ 10 read counts at rs16969968 in both DNA-WGS and RNA-seq were used to analyze allelic expression imbalance (AEI). To minimize technical or copy-number-associated bias (tumor-specific deletions or amplifications) that might affect allelic ratios, we repeated the analysis in samples with DNA rs16969968-A allelic ratios close to 0.5 (0.4–0.6, *n* = 58). Differences in rs16969968-A allelic ratios between RNA and DNA of each individual were assessed with the paired Wilcoxon test and across smoking groups (never, former, and current smokers) with the unpaired Kruskal–Wallis test.

### In silico splicing and RNA-binding protein analyses

Putative donor and acceptor splice sites within *CHRNA5* exons 4–6 and corresponding introns 4 and 5 were predicted with the Alternative Splicing Site Predictor [18]. Binding sites for RNA-binding proteins (RBPs) within *CHRNA*5-exon 5 were predicted using the online tools RBPDB (http://rbpdb.ccbr.utoronto.ca/) [19] and RBPmap (https://rbpmap.technion.ac.il/index.html*)*, with default settings. The sequences were analyzed with both the rs16969968-A and G alleles. Linkage disequilibrium (LD) analysis of all genetic variants within *CHRNA5* introns 4 and 5 and exon 5 was performed using LDlink (https://ldlink.nih.gov/) in 2504 individuals from the 1000 Genomes Project to identify variants correlated (r^2^) with rs16969968. The same genomic region was also explored in 452 human long-read genome assemblies from 226 individuals, as previously described [16] to search for potentially novel genetic variants that might affect observed splicing patterns.

### In vitro model of smoking exposure

To model smoking exposure, cells were cultured in medium with cigarette smoke concentrate (CSC; Murty Pharmaceuticals #NC1560725). CSC represents the total particulate matter of cigarette smoke collected by passage through a filter and dissolved in DMSO before use. The CSC concentration and treatment duration were selected to approximate the exposure levels reported for chronic smokers [20–22]. The default treatment included 25 µg/mL CSC in DMSO, and DMSO-only (vehicle control), with a final DMSO concentration of 0.0625% in both treatment conditions. For short-term treatment, the cells were treated under default conditions for 48 h. To model chronic exposure, cells designated “long-term CSC-treated” (LT-CSC) or “long-term DMSO-treated” (LT-DMSO) were continuously treated for > 30 days, then cryopreserved or maintained under the same treatment conditions. For experiments, previously cryopreserved LT-CSC and LT-DMSO cells were thawed and replated. Cells were continuously cultured under default conditions (for RT-qPCR minigene and AEI assays) or additionally treated for 7 days with 50 µg/mL CSC and 0.125% DMSO before transfection (for long-read minigene cDNA sequencing). Under these conditions, no adverse effects on cell viability were observed, which is consistent with prior reports [21].

### RNA sequencing and splicing analysis of endogenous *CHRNA5*

Total RNA was isolated from cells in three biological replicates per condition. High RNA quality was verified (RNA integrity number equivalent, RINe > 9.0) with ScreenTape (Agilent). Libraries were prepared from 200 ng of input RNA using the KAPA RNA HyperPrep with RiboErase rRNA depletion and indexed with xGen Dual Index UMI Adapters (IDT). Multiplexed libraries (250–350 bp inserts) were sequenced on the Illumina NovaSeq 6000 platform (paired-end, 150 bp; 279–418 million reads/sample). Sequencing was performed by the Cancer Genomics Research Laboratory (DCEG/NCI).

Quality control and base trimming of the RNA-seq reads were performed with Partek Flow. The processed reads were aligned to the GRCh38 reference genome with STAR (v2.7.8a) [23]. Differential gene expression analysis was conducted with DESeq2 using the Partek E/M quantification method and ENSEMBL release 111 transcript annotations, with DMSO-treated samples used as the reference group. Gene Ontology (GO) and gene set enrichment analysis (GSEA) were performed on the DEGs via the Kyoto Encyclopedia of Genes and Genomes (KEGG) database.

The aligned transcriptomes were visualized with IGV (v2.19.4). The quantification of splice junctions for all *CHRNA5*-exon 5 isoforms was performed using FeatureCounts (v4.5.0, in R) and a custom .bed file, which provided coordinates for each isoform-specific junction. Only samples with ≥ 30 total exon 5 splice junction reads were retained for analysis (A549: *n* = 3, UMUC3: *n* = 2; 67–211 junctions per sample); this excluded one UMUC3 replicate with insufficient junction read counts. Isoform ratios were calculated as the fraction of specific junctions over total junction reads. For each cell line, differences in isoform ratios between LT-CSCs and LT-DMSO were compared by unpaired two-sided Student’s t-test; *p* < 0.05 was considered significant.

Raw RNA-seq reads from a neuroblastoma cell line (SH-SY5Y) with or without retinoic acid (RA) treatment [24] were obtained from the SRA NCBI (BioProject PRJEB44501). The RNA-seq reads were aligned to the human genome assembly GRCh38 using the STAR aligner (v2.7.10b) and deduplicated using Picard MarkDuplicates (v2.26.11). The resulting BAM files were sorted and indexed with Samtools (v1.21) and used for isoform exon-junction quantification as described above.

Transcripts per million (TPMs) in the A549 and UMUC3 RNA-seq datasets were calculated from raw read counts generated by Partek Flow using standard TPM normalization (counts normalized to gene length and library size, scaled to per million). For the SH-SY5Y RNA-seq dataset, TPMs were calculated by RSEM v1.3.3 with default parameters for alignment and quantification against the human genome assembly GRCh38.

### Exontrap-*CHRNA5*-exon5 minigene construction

A 2,823-bp fragment containing *CHRNA5*-exon 5 (832 bp) flanked by intronic sequences (865 bp upstream and 1,126 bp downstream) was PCR-amplified from human germline DNA (1000 Genomes Project sample HG3540 purchased from Coriell Institute, rs16969968-G/G genotype) using Q5 HotStart Master Mix (NEB) and primers with sites for the restriction enzymes XhoI and SacII (Table S1). The PCR product and Exontrap vector pET01 (MobiTec) were digested with XhoI and SacII (NEB), ligated, and transformed into *E. coli* competent cells (stbl3, Invitrogen). Colonies were PCR-screened with Exontrap Primer02 and Primer03, and the insert size (3.6 kb) was validated using a D5000 TapeStation (Agilent). Inserts were confirmed by Sanger sequencing, and plasmid DNA was purified using an endotoxin-free Maxiprep Kit (Qiagen). The rs16969968-G allele in the Exontrap-*CHRNA5*-exon5 minigene was replaced by the A allele using site-directed mutagenesis (QuikChange II, Agilent) and primers listed in Table S1. The sequence was verified by whole-plasmid sequencing (6,922 bp, Oxford Nanopore, Eurofins), confirming the substitution with no additional sequence variants detected.

### Transfection

Cells were seeded in 6-well plates 24 h before transfection at 2 × 10^5^ cells/well. A549 and UMUC3 were transfected with 2.5 µg of Exontrap-*CHRNA5*-exon5 minigene DNA using Lipofectamine 3000 (Invitrogen) in biological triplicates and quadruplicates, respectively. Controls included an empty Exontrap vector and a GFP-expressing plasmid (mEGFP-N1; Addgene #54767). The medium was replaced with fresh treatment medium 4 h post-transfection (for long-term treatment), or cells were treated immediately before transfection and maintained in the same medium (for short-term treatment); the cells were harvested for RNA extraction 48 h post-transfection. Transfection efficiency was assessed using the pmaxGFP control plasmid (Lonza) and was ~ 50% in both A549 and UMUC3.

### RNA extraction

The cells were lysed in 350 µl of RLT buffer (Qiagen) supplemented with β-mercaptoethanol. Total RNA was isolated using the RNeasy Mini Kit with on-column DNase I digestion. RNA quantity and purity were assessed using a NanoDrop 8000 (Thermo Fisher Scientific).

### Oxford Nanopore targeted long-read sequencing of minigene-derived cDNA

Minigene-specific cDNA was synthesized in 20 µl reactions via SuperScript III First-Strand Synthesis (Invitrogen) and a vector-specific primer (Exontrap Primer01, Table S1). The resulting cDNA was amplified by RT-PCR using Exontrap Primer02 and Primer03 with NEB Q5 HS Master Mix. The RT-PCR products were resolved on 1.2% agarose gels. The expected bands included 849 bp (unspliced empty vector) and 1,078 bp (full-length *CHRNA5*-exon 5 spliced with vector exons). Additional bands were considered as evidence of potential alternative splicing. The gel images were captured on a Bio-Rad ChemiDoc system, and the band intensities were quantified with Image Lab software.

Amplicons for Oxford Nanopore Technologies (ONT) sequencing were generated by RT-PCR as described above, with the same primers modified to include ONT adapter sequences. Libraries were prepared with the ONT “Ligation Sequencing V14 – PCR Barcoding” protocol (SQK-LSK114 with EXP-PBC001 or EXP-PBC096). The PCR products from all the experimental replicates were barcoded, pooled at equimolar ratios (by size and mass, Agilent TapeStation and Qubit, respectively), and sequenced on an R10.4.1 PromethION flow cell using the PromethION 2 Integrated (P2i) device for 72 h (ONT MinKNOW, high-accuracy basecalling).

The reads were aligned to a custom reference sequence comprising the vector and amplicon using minimap2 within ONT EPI2ME workflows. Isoforms were classified based on the last amino acid within *CHRNA5*-exon 5 retained after splicing to exon 6 (e.g., “R372”). Splice junctions were quantified using FeatureCounts (v4.5.0, in R) and a .saf file, which provided coordinates for each isoform-specific junction. Isoform ratios were calculated as individual isoform junctions relative to all exon 5 isoform junctions per sample. Treatment group differences were assessed by a non-paired, two-sided Student’s t-test (*p* < 0.05).

### RT‒qPCR analysis of minigene splicing

Alternative splicing of the Exontrap-*CHRNA5*-exon5 minigene was evaluated by custom TaqMan qPCR assays (Table S1) individually targeting all splicing isoforms; a reference assay targeting the junction between the upstream vector exon and the cloned *CHRNA5*-exon 5 was used for normalization in each sample. RNA was extracted as described above, and cDNA was synthesized from 1 µg of total RNA/sample in 20 µl reactions with the iScript Reverse Transcription Supermix (Bio-Rad). The cDNA was diluted with 80 µl DI water, resulting in an input RNA concentration of 10 ng/µl. TaqMan assays were performed in technical quadruplicate in 384-well plates on a QuantStudio 7 Flex Real-Time PCR System (Applied Biosystems), with 2 µl of cDNA, corresponding to 20 ng RNA input per 5 µl reactions.

The correlation coefficients and amplification efficiencies for all the TaqMan assays were experimentally determined to be comparable (78.3–95.7%, Table S1) based on six serial ×2 dilutions of the cDNA generated from one sample of A549 cells transfected with the Exontrap-*CHRNA5*-exon 5 minigene. The templates included cDNA for the minigene or empty vector, as well as negative controls (water or minigene DNA). The expression levels (average Ct values for technical quadruplicates) of each assay/sample were normalized to the Ct values of the reference assay to generate ΔCt values for each sample. Comparisons between CSC- and DMSO-treated samples were performed as ΔΔCt = ΔCt CSC - ΔCt DMSO, and the relative fold difference between the treatment groups was calculated as fold = 2^− ΔΔCt^.

### Analysis of Allelic Expression Imbalance (AEI) in SH-SY5Y cells

Bioinformatically, AEI was evaluated as the ratios of the rs16969968-A allele in genomic DNA (WGS, CCLE) and in cells treated with control or retinoic acid (RNA-seq, SRA BioProject PRJEB44501 [24]). Experimentally, AEI was evaluated using an allele-specific TaqMan genotyping assay that quantified the rs16969968-A and rs16969968-G alleles in genomic DNA and cDNA from SH-SY5Y cells subjected to short- or long-term treatment with CSC or DMSO, in 6 biological replicates, as described above. For genotyping, positive controls included DNA from cells with known rs16969968 genotypes (A549-G/G, UMUC3-A/A, and SH-SY5Y-A/G; Figure S1), and negative controls included water, no-Reverse Transcriptase, and no-RNA input reactions. Genotyping was performed using a commercial assay for rs16969968 (C__26000428_20, Thermo Fisher, with VIC and FAM fluorophores corresponding to the A and G alleles, respectively) and Type-it Fast SNP Probe Master Mix (Qiagen), in 4 technical replicates. To compensate for potential technical differences due to allelic probe compositions and VIC and FAM fluorescence intensities, the A: G ratio in gDNA was assumed to be 50:50% and used to normalize cDNA values [25]. Ct values for the rs16969968-A and G alleles in cDNA samples were averaged across technical replicates (*n* = 4) and normalized to the Ct value of the corresponding gDNA, averaged across 2 samples with 4 technical replicates each, generating normalized ΔCt values. The allelic expression in each sample was estimated as 2^− ΔCt^. AEI was calculated as the proportion of rs16969968-A allele expression relative to total allelic expression, A/(A + G). Differences in the rs16969968-A allelic ratios between groups were evaluated using unpaired two-sided Student’s t-tests.

## Results

### Exon 5 of *CHRNA5* is a splicing hotspot

Several *CHRNA5* isoforms are listed in public databases (NCBI and ENSEMBL, Table S2). Five of these isoforms, all generated by alternative splicing of the largest exon of this gene (exon 5, 832 bp), have been reported and partially annotated [13] (Table S2). Although *CHRNA5* expression is ubiquitous, it is relatively low, with median transcripts per million (TPM) values in normal human tissues from the Genotype-Tissue Expression (GTEx) project ranging from 0.042 (liver) to 3.01 TPM (EBV-transformed lymphocytes, Figure S2).

**Fig. 1:**
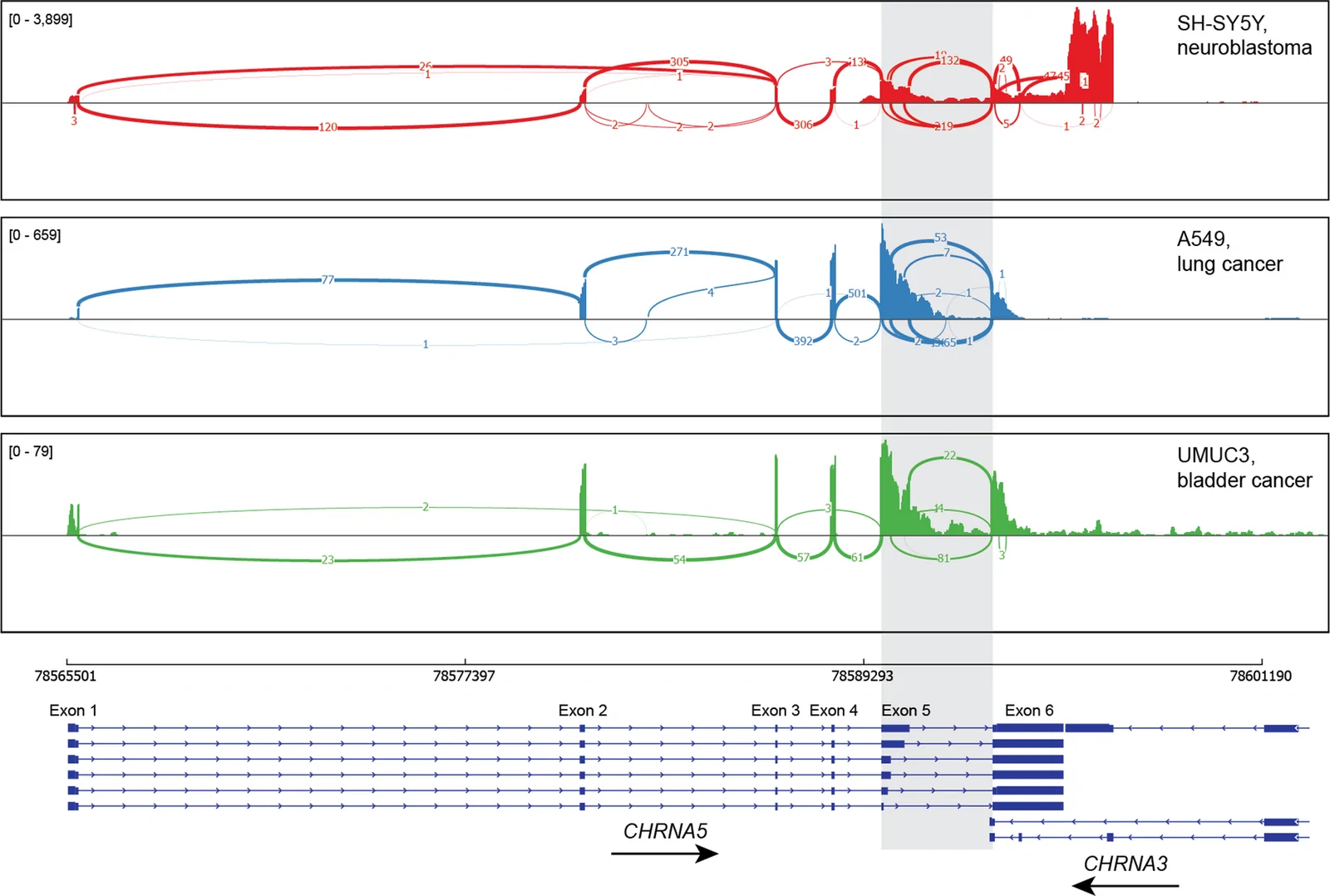
Splicing analysis of *CHRNA5* based on RNA-seq in the human cell lines SH-SY5Y, A549, and UMUC3. Splicing of *CHRNA5* is predominantly limited to events between exons 5 and 6, generating six main isoforms. *CHRNA5* splicing patterns were visualized via Sashimi plots with the Integrative Genomics Viewer (IGV). Data sources: SH-SY5Y (SRA BioProject PRJEB44501), A549 and UMUC3 (current work), all at untreated baseline conditions. The representative isoform ratios are presented in Table S4

**Fig. 2:**
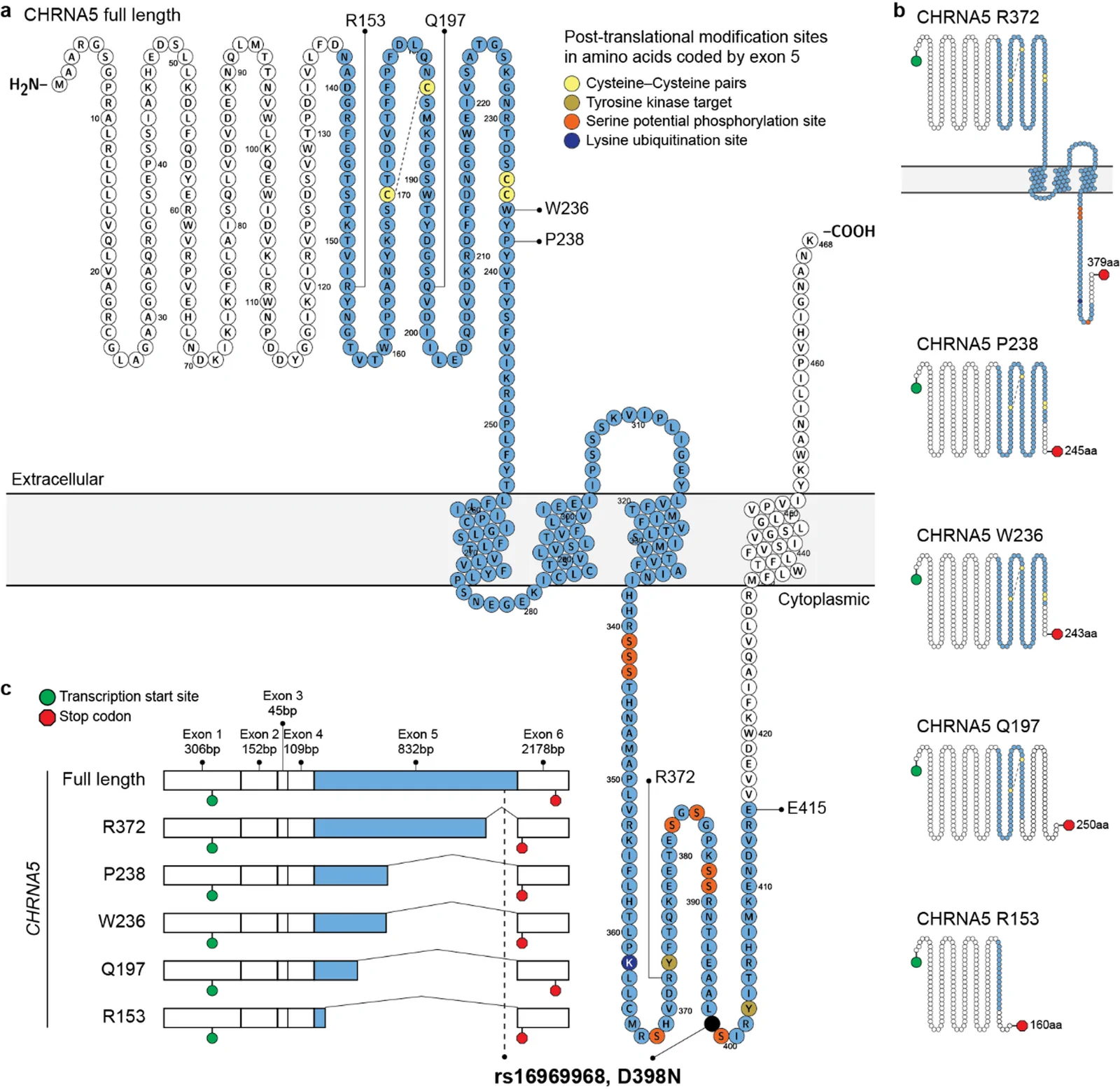
*CHRNA5* alternative transcripts and protein products. Schematic of **a** the full-length (FL) and **b** five main alternatively spliced isoforms of *CHRNA5* and their protein products. Protein domains were annotated according to UniProtKB (accession P30532), with numbering based on the FL protein: extracellular domain (ECD), 23–254 aa and 452–468 aa; transmembrane domain 1 (TMD1), 255–275 aa; TMD2, 282–302 aa; TMD3, 317–337 aa; TMD4, 430–451 aa; and the intracellular domain (ICD), 338–429 aa. Key structural features are marked: Cys-Cys loop (C170, C184) within the ECD; rs16969968 (D398N), lysine ubiquitination site (K362), and tyrosine residues (Y373, Y402) within the ICD. Splicing isoforms are labeled based on the last retained residue of exon 5. Protein topology was visualized with Protter v1.0. Two additional minor isoforms with internal splicing are annotated in Figure S3

Thus, we explored *CHRNA5* splicing in several human cell lines with robust *CHRNA5* expression: A549 (lung adenocarcinoma; DepMap TPM = 47.5), SH-SY5Y (neuroblastoma; TPM = 20.3), and UMUC3 (bladder carcinoma; TPM = 11.2). RNA-seq analysis of these cell lines (Fig. 1) confirmed that alternative splicing was largely restricted to exon 5. In addition to the full-length (FL) reference isoform, we observed five main alternative isoforms, each lacking 130–786 bp of exon 5, and truncating the CHRNA5 protein by 89–309 aa (Fig. 2). Splicing sites for all *CHRNA5*-exon 5 isoforms were also predicted by bioinformatics analysis and were not affected by rs16969968 genotypes (Table S3). We also observed two uncommon internally spliced isoforms, in which 122–128 bp were excluded, truncating the protein by ~ 228–230 aa (Figure S3, Table S3). Because exon 5 harbors the functional rs16969968 variant (A > G, D398N), which is strongly associated with smoking behavior and cancer risk [7–9], we further explored splicing events affecting this exon in three cell lines, which represent all rs16969968 genotypes: A549 (G/G), SH-SY5Y (A/G), and UMUC3 (A/A) (Figure S1). Analysis of human long-read genome assemblies [16] did not identify any genetic variants underlying the alternative splicing sites.

In A549 and UMUC3 cell lines, the full-length (FL) isoform accounted for ~ 45–49% of all *CHRNA5*-exon 5 junctions (Table S4, Figure S4, DMSO control group). In SH-SY5Y cells (Table S4, Figure S5a), the FL isoform ratio was greater (71.6% at baseline) and further increased to 79.9% (*p* = 0.034) after treatment with retinoic acid [24], which induced the differentiation of neuroblast-like to neuronal-like cells, suggesting the dynamic nature of alternative splicing of this exon.

**Fig. 3:**
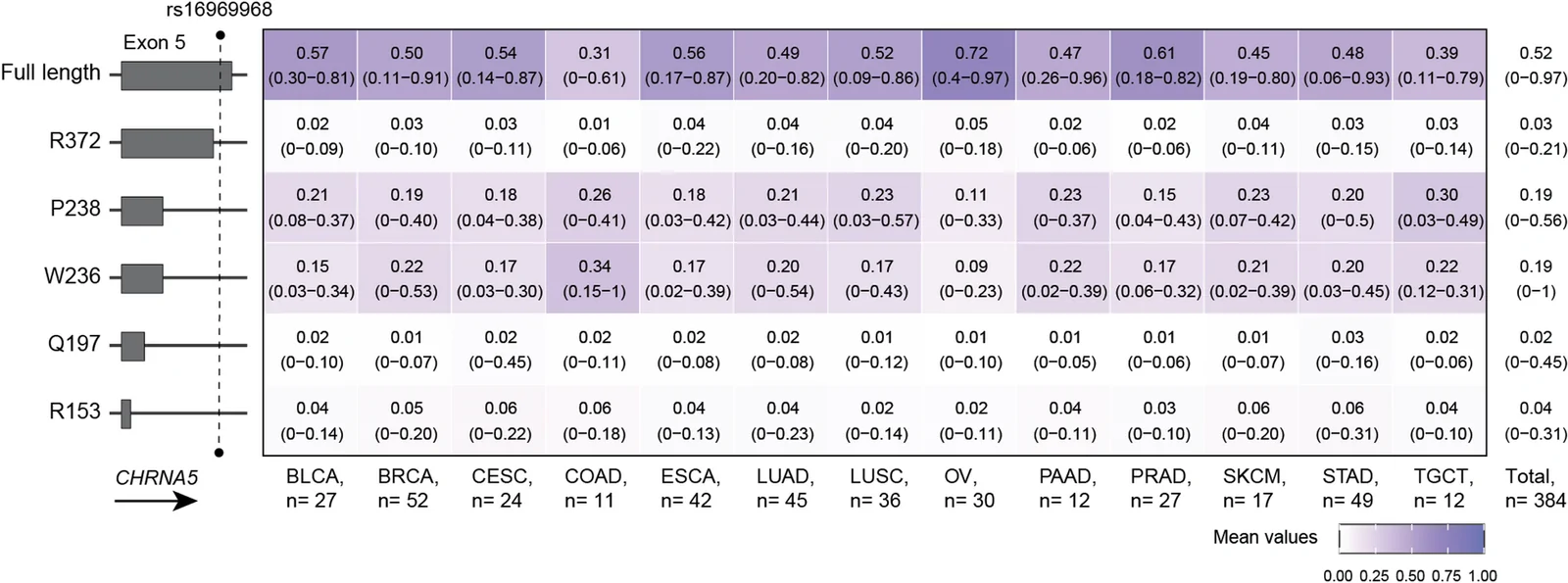
Ratios of the six splicing isoforms involving exon 5 of *CHRNA5* in TCGA tumors. Analysis is limited to tumor types with ≥ 10 samples with ≥ 30 *CHRNA5*-exon 5 splice junctions per sample. Sample counts are shown for each tumor type and the total for all tumors; ratios are shown with mean, minimum and maximum values. The position of rs16969968-D398N, which is missing in all splicing isoforms, is marked. The full results are provided in Table S5

To comprehensively characterize *CHRNA5*-exon 5 splicing in human tissues, we analyzed RNA-seq data for tumors from The Cancer Genome Atlas (TCGA) and normal tissues from the GTEx project. At a threshold of ≥ 10 samples per tissue with ≥ 30 junction reads across five main annotated exon 5 isoforms per sample (the minor isoforms with internal splicing were not annotated). Based on these criteria, 384 tumors from 13 TCGA cancer types were selected, but no GTEx samples. In TCGA tumors, the FL isoform comprised 52.4% (range 31.2–72.0%) of all exon 5 junctions, followed by isoforms P238 (19.7%, range 11.3–30.3%), W236 (18.7%, 9.2–34.0%), and the less abundant isoforms R153 (4.3%, 2.2–6.2%), R372 (3.2%, 1.4–4.5%), and Q197 (1.7%, 0.06–3.4%) (Fig. 3 and Table S5). Thus, in both human cell lines and tumors, exon 5 represents a splicing hotspot within *CHRNA5*.

### Cigarette smoke concentrate (CSC) as a model of smoking exposure in cell lines

The observed splicing variability in *CHRNA5*-exon 5 suggested that multiple factors, including smoking, may regulate it. Stratification by patient smoking status was not feasible in our TCGA-based splicing analysis (Fig. 3) due to relatively low *CHRNA5* expression, which resulted in small sample sizes of each tumor subset, a predominance of smokers among cancer patients, and limited data on smoking intensity and duration.

To directly test the impact of smoking on exon 5 splicing, we treated cells with cigarette smoke concentrate (CSC), a total particulate matter isolated from cigarette smoke [26–29]. Based on the literature [20–22], we used 25 µg/mL CSC in DMSO or DMSO as a vehicle control. Continuous treatment of A549 and UMUC3 cells for 30 days generated corresponding paired long-term (LT)-treated cell lines (LT-DMSO and LT-CSC).

RNA-seq profiling of these cell lines identified differentially expressed genes (DEGs), including several well-known smoking-responsive genes, such as *CYP1A1/B1*, which encode members of the cytochrome P450 family, and *AHRR*, which encodes the aryl hydrocarbon receptor repressor [30–33]. Both genes were significantly induced in A549 cells but not in UMUC3 cells, where their expression was low (Table S6). Notably, *CHRNA5* expression was not affected by CSC treatment in either cell line (Table S7, Table S8).

In A549 LT-CSC cells compared with the LT-DMSO control, 4,556 DEGs were identified based on nominal significance (*p* < 0.05), and 2,328 of those DEGs were significant at FDR < 0.05 (Table S9). Pathway analysis revealed enrichment for smoking-related processes, including KEGG:05207 Chemical carcinogens – receptor activation (FDR = 1.65E-2; Table S9) and GO:0042221 Response to chemical (FDR = 2.02E-15; Table S10). In UMUC3-LT cells, 341 DEGs were identified at *p* < 0.05, but only four met FDR < 0.05 (Table S8), which was insufficient for pathway analysis. Overall, these results confirmed that our CSC model recapitulates the expected transcriptomic signatures of smoking exposure.

We then queried *CHRNA5*-exon 5 splicing ratios in the A549-LT and UMUC3-LT RNA-seq datasets (Figure S4), as in the TCGA analysis (Fig. 3). The splice junction counts for exon 5 in our experiments were modest – for total counts of all isoforms (means: 170.3 in A549 and 54.8 in UMUC3), and for the most abundant isoform, FL (means: 81.7 and 23.5, respectively), without statistically significant differences by treatment conditions (Table S4). Based on these results, we conclude that models with higher endogenous *CHRNA5* expression/sequencing coverage, or minigene assays, would be more informative for detecting CSC-induced splicing changes in this gene.

### CSC treatment alters the splicing of the Exontrap-*CHRNA5-*exon5 minigene

To explore *CHRNA5*-exon 5 splicing under controlled conditions, we engineered an Exontrap-*CHRNA5-*exon5 minigene by cloning the complete exon 5 (832 bp) with its flanking intronic sequences into Exontrap, a eukaryotic reporter designed to evaluate splicing events. The minigene was transfected into the LT-DMSO and LT-CSC cell lines A549 (*n* = 3) and UMUC3 (*n* = 4) for 48 h. The splicing products of the minigene were first assessed by RT-PCR using vector-specific primers flanking the insert. A 1,078 bp band corresponded to the FL exon 5 isoform, whereas longer and shorter bands represented intron retention and exon 5 truncations, respectively. RT-PCR and agarose gel visualization of the products revealed the range of the minigene splice products (Figure S6).

**Fig. 4:**
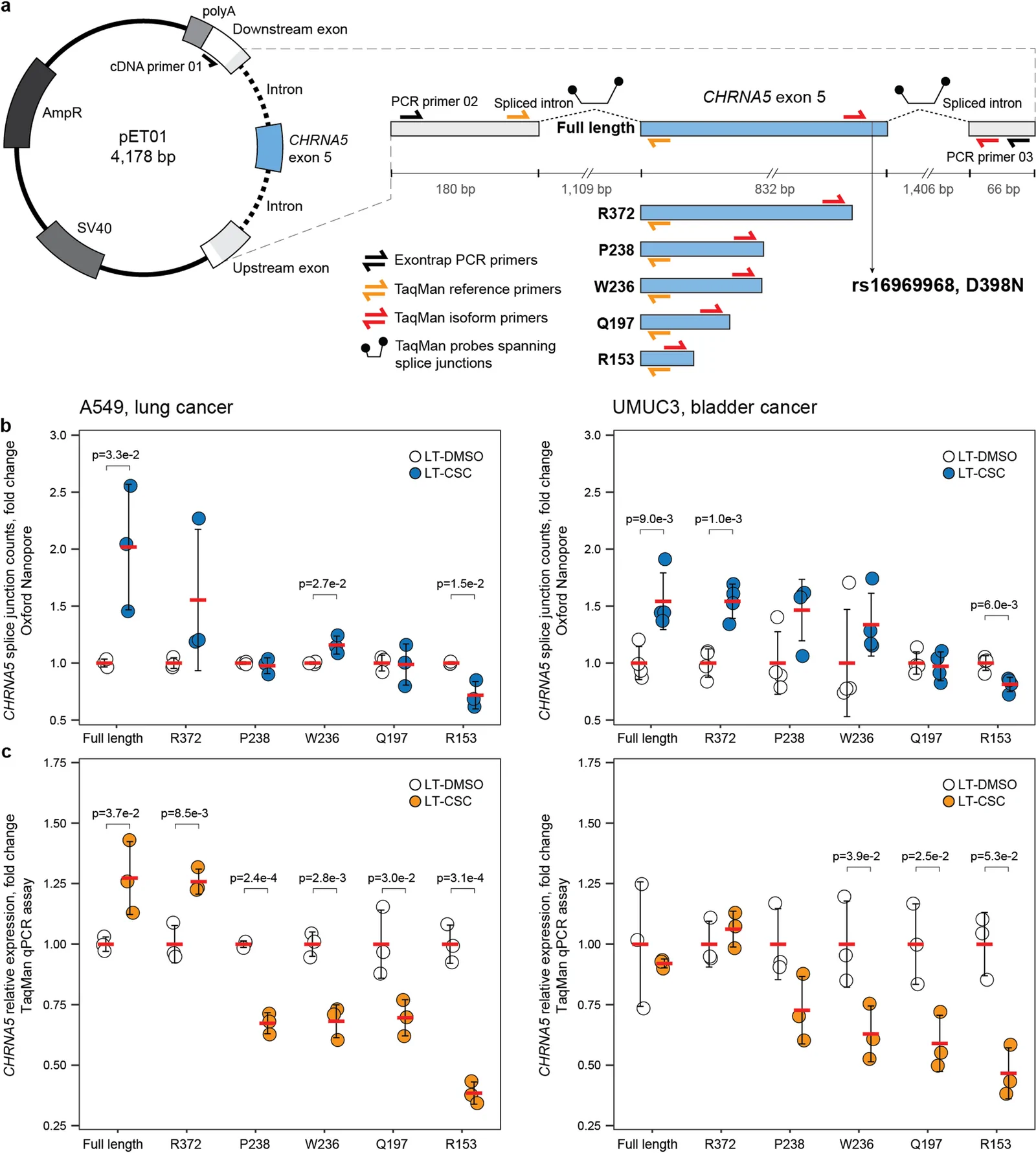
Analysis of splicing patterns of Exontrap-*CHRNA5*-exon5 minigene in A549 and UMUC3 cell lines. **a** Schematic of the Exontrap vector pET01 with *CHRNA5-*exon5 cloned between vector exons. Positions of PCR and TaqMan primers/probes are indicated. Fold differences in splicing ratios of the six main *CHRNA5-*exon 5 isoforms between long-term CSC- and DMSO-treated A549 (*n* = 3) and UMUC3 (*n* = 4) cells transfected with Exontrap-*CHRNA5*-exon5 minigene were quantified by **b** long-read targeted cDNA sequencing (Oxford Nanopore) and **c** isoform-specific TaqMan expression assays. Individual data points represent biological replicates, with means (red lines) and standard deviations (error bars). P-values are for two-sided Student’s t-tests. Splicing patterns of Exontrap-*CHRNA5*-exon5 minigenes with the rs16969968-G and A alleles in A549 cells are shown in Supplementary Figure S8

The minigene-specific transcripts were quantified by targeted long-read cDNA sequencing with Oxford Nanopore Technologies (ONT). Multiplexed libraries were generated separately for A549 (3 replicates) and UMUC3 (4 replicates), each combining LT-CSC, LT-DMSO, and empty-vector controls. Because ONT sequencing of PCR amplicons can be biased toward shorter fragments, relative isoform ratios were compared between, rather than within, treatment groups. In both cell lines, LT-CSC treatment significantly increased the FL isoform ratio and decreased the shortest isoform ratio (R153). Additionally, W236 was enriched in A549 LT-CSC cells, and R372 was enriched in UMUC3 cells (Fig. 4). Because the internal splicing isoforms W236-G279 and P238-G279 represented only < 2% of all endogenous *CHRNA5*-exon 5 junction reads (Table S3), these isoforms were considered minor and not evaluated further.

For orthogonal validation, we performed isoform-specific TaqMan RT-qPCR assays in samples generated in independent experiments. The ONT and TaqMan results were generally consistent, with relative ratios of shorter isoforms decreasing and those of longer isoforms increasing. Specifically, a decrease in the R153 ratio following CSC treatment was detected in both cell lines using both detection methods, and an increase in the ratios of the FL and R372 isoforms was observed in A549 cells (Fig. 4, Table S11). In contrast with long-term CSC treatment (> 30 days) of A549 cells, Exontrap-*CHRNA5-*exon5 minigene splicing was not significantly affected by short-term treatment (48 h, Figure S7, Table S11). Similar isoform splicing patterns were observed for the Exontrap-*CHRNA5-*exon5 minigenes with rs16969968-A and -G alleles in A549 cells (Figure S8, Table S11).

The prediction of binding sites for RNA-binding proteins (RBPs) identified 7 RBPs with 20 binding sites within the FL and R372 isoforms (Table S12). Among those RBPs, *A2BP1/RBFOX1* and *RBMY1A1* were not expressed in our RNA-seq datasets of A549 and UMUC3 cells, whereas *FUS*, *SAP49/SF3B4*, *PUM2*, *SFRS9*, *MBNL1*, and *eIF4B* were expressed but not altered by CSC treatment of A549 or UMUC3 cells (Table S7, Table S8). Although expression of RBPs with predicted binding sites within *CHRNA5* exon 5 (such as *FUS* or *PUM2*) did not change by CSC treatment, post-translational modifications of the corresponding proteins, as well as their role in *CHRNA5* splicing, might be affected.

No RBPs were predicted to bind to 50-bp sequences centered on either rs16969968-A or -G alleles. Within *CHRNA5* sequences included in the minigene (intron 4-exon 5-intron 5), no new genetic variants were identified. Of the known variants, only 4 SNPs showed moderate correlation with rs16969968 (r^2^ range of 0.23–0.30 in European populations and lower in other populations). One SNP is located in intron 4 and three in intron 5 (Table S13), and thus cannot account for splicing changes observed within exon 5 of *CHRNA5*.

### *CHRNA5-*exon 5 splicing events are independent of rs16969968

Bioinformatics analysis showed no effect of the rs16969968 alleles on *CHRNA5*-exon 5 splicing (Table S3). Furthermore, analysis of endogenous *CHRNA5* splicing under CSC/DMSO treatment conditions showed results similar to those in cell lines with the rs16969968-G/G (A549) and rs16969968-A/A (UMUC3) genotypes, and comparable to those in the Exontrap minigene carrying the G or A allele (in A549 cells, Figure S8). If cigarette smoke exposure altered exon 5 splicing preferentially in transcripts carrying the rs16969968-A (risk) or G (reference) allele, which are eliminated from the resulting alternative splicing isoforms, such effects would be detectable in heterozygous samples as allelic expression imbalance (AEI).

**Fig. 5:**
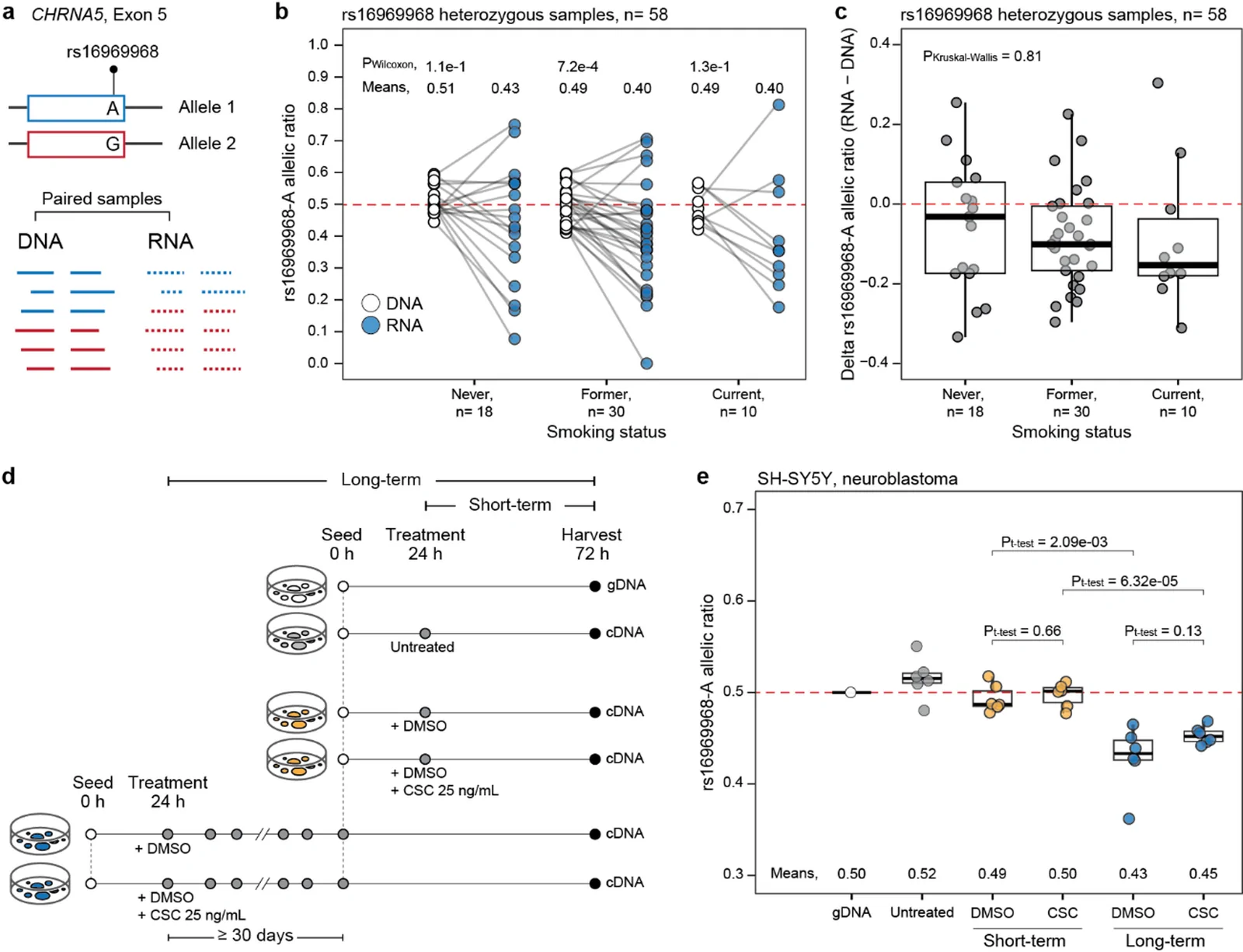
Analysis of allelic expression imbalance (AEI) in 58 BLCA-TCGA tumors and SH-SY5Y cells. **a** Schematic of the allelic expression imbalance (AEI) concept. **b** The ratio of the rs16969968-A risk allele in paired DNA and RNA samples from the same tumors, stratified by smoking status (never, former and current). Each line represents one sample. The differences in allelic ratios between DNA and RNA were evaluated using a paired Wilcoxon test. **c** Differences in the rs16969968-A allele ratios between RNA and DNA (delta = RNA ‒ DNA) in each sample across smoking groups. Samples were restricted to those with sufficient coverage in DNA and RNA (> 10 reads) and DNA allelic ratios close to 0.5 (0.4–0.6). The differences in deltas across smoking groups were evaluated with the Kruskal‒Wallis test. Similar results were obtained in the full set of heterozygous samples (*n* = 101 DNA/RNA paired samples, *p* = 0.81). **d** Schematic of the AEI evaluation in SH-SY5Y neuroblastoma cells, showing the ratio of the rs16969968-A allele in genomic DNA, after short-term (48 h) and long-term continuous treatment (≥ 30 days) with vehicle control (DMSO) or CSC (25 ng/mL). **e** Expression ratios of the rs16969968-A allele in cDNA in indicated conditions, with the ratio in genomic DNA set to a baseline of 0.50. Individual data points represent biological replicates, with boxes indicating the interquartile range and the median (line). P-values are for two-sided Student’s t-tests. Full results are presented in Table S14

To assess this, we analyzed the BLCA-TCGA cohort of bladder cancer patients. Among those, germline genotyping of blood-derived DNA identified 101 samples heterozygous for rs16969968, with sufficient coverage in both DNA and RNA samples from the same tumors. For each patient, we calculated rs16969968-A risk allele ratios to assess whether the region containing rs16969968 was preferentially spliced out of transcripts carrying either allele. The risk rs16969968-A allele was less frequent in RNA than in paired DNA samples, consistent with previous reports that this allele resides on a haplotype associated with decreased *CHRNA5* expression, likely due to transcriptional regulation [34]. However, we found no significant differences in the rs16969968 allelic ratios in samples stratified by smoking status (never, former, and current smokers) (Fig. 5).

Similarly, we investigated AEI in the SH-SY5Y cell line with the rs16969968-A/G genotype. Treatment with retinoic acid induced neuronal differentiation in these cells and shifted the ratio of *CHRNA5* splicing isoforms (Figure S5a), but without changing the rs16969968-A allelic ratio (Figure S5b). We also experimentally tested the effect of CSC/DMSO in this cell line, evaluating AEI by direct genotyping of rs16969968; however, we observed no evidence of differential AEI with either short- or long-term CSC treatment (Fig. 5, Table S14). We noted, however, that the rs16969968-A risk allelic ratio decreased compared to untreated cells (0.52) with long-term treatment with either DMSO (0.43) or CSC/DMSO (0.45), whereas short-term treatment did not have this effect. These results indicate a similar probability of smoking-induced splicing events in *CHRNA5* transcripts with either the rs16969968-A or G allele, despite potential cellular adaptation to long-term DMSO treatment affecting the rs16969968-A allelic ratio.

## Discussion

In this study, we present experimental evidence supporting a model in which cigarette smoking shapes the splicing landscape of *CHRNA5*, thereby altering the structural composition and functional properties of α5-containing nAChRs, with downstream consequences for nicotine dependence, tumor biology, and cancer risk.

Alternative splicing of *CHRNA5* predominantly involves its largest coding exon, exon 5 (832 bp). Analysis of RNA-seq data from 384 tumors of 13 types in TCGA demonstrated that only 52.4% of all exon 5 spanning splice junctions corresponded to the FL isoform. A similar ratio (45–49%) was observed in cell lines representing smoking-related cancers - A549 (lung) and UMUC3 (bladder). In the neuroblastoma cell line SH-SY5Y, this ratio was 71.6% at baseline and shifted to 79.9% after neuronal differentiation, demonstrating the dynamic nature of splicing events under different cellular conditions.

We hypothesized that smoking exposure might affect splicing in exon 5, and tested this hypothesis in A549 and UMUC3 cells treated with cigarette smoke concentrate (CSC), which contains nicotine and other tobacco combustion products. Because this analysis required high coverage of splice junctions, which was insufficient given endogenous expression in A549 and UMUC3 cells in our experiments, we used an engineered splicing reporter, the *CHRNA5*-exon 5-minigene, transiently expressed in cells. Splicing of this minigene was profiled using targeted Oxford Nanopore long-read cDNA sequencing and isoform-specific TaqMan assays. Long-term CSC (for at least 30 days) but not short-term (48 h) treatment resulted in statistically significant, isoform-specific shifts in splicing ratios, generally decreasing the ratio of the shortest isoforms and increasing the ratio of the longest isoforms, demonstrating that constituents of cigarette smoke can directly modulate *CHRNA5* splicing.

We investigated six *CHRNA5* isoforms, including five with 130–786 bp deletions in exon 5. Five of those (all except R372) have been previously characterized in A549 cells [13], with Western blotting confirming the expression and cell-surface localization of the FL α5 protein (isoform 1; 468 aa) and three truncated variants: P238 (isoform 3, − 233 aa), W236 (isoform 2, − 225 aa), and Q197 (isoform 4, − 218 aa). The shortest isoform, R153 (isoform 5, − 308 aa), was undetectable, likely due to its instability [13]. The longest alternative variant, R372, has not been studied previously, but given its minimal truncation (89 aa), it is predicted to be more stable than shorter isoforms.

Mapping exon 5 splicing events onto the CHRNA5 domain architecture revealed extensive disruption of functionally critical regions. Exon 5 encodes part of the extracellular (EC) domain, including the conserved cysteine loop (C170–C184) [10]. This disulfide bond undergoes conformational rearrangement upon agonist binding and is crucial for channel gating [10, 35]. Only R153 lacks this motif, rendering it nonfunctional. Exon 5 also encodes three of the four transmembrane (TM) domains, TM1–TM3. Except for R372, all alternative isoforms lack these domains, likely impairing membrane integration, subunit assembly, and receptor stability [10]. R372 retains TM1–TM3 but lacks TM4 (encoded by exon 6), whereas Q197 retains TM4 only. The long cytoplasmic loop between TM3 and TM4, representing the intracellular domain (ICD), is encoded primarily by exon 5 (92 aa; residues 338–429). This domain, which is partially retained only in R372, modulates assembly, trafficking, protein‒protein interactions, desensitization, and turnover [36, 37].

Loss of ICD residues due to exon 5 splicing also affects post-translational modification sites that are important for receptor trafficking, membrane localization, and turnover [36]. The FL CHRNA5 protein includes nine serines (potential phosphorylation sites), which are partially lost with splicing; two tyrosines (tyrosine kinase targets) are lost in all alternative isoforms; and one lysine (K362), the sole site for the ubiquitin-proteasome system (UPS) targeting via ER-associated-degradation (ERAD), is lost in all but the R372 isoform. Loss of several phosphorylation sites is predicted to alter receptor regulation and signal transduction. Loss of K362 abolishes the primary degradation signal, potentially allowing defective subunits to evade quality control and incorporate into receptors, especially if UPS activity is suppressed. Most nAChRs assemble intracellularly, with 65–85% stored in intracellular pools, and up to 80% undergo degradation if incorrectly assembled or unused [10]. A reduction in proteasomal activity due to CSC and chronic nicotine exposure can increase surface nAChR levels up to fourfold [38–40].

Thus, truncated CHRNA5 variants, if stable enough, could integrate into α5-nAChRs that accumulate at the membrane. Cell surface turnover of these defective receptors could be decreased by impaired ERAD engagement due to splicing-related loss of K362, as well as inhibition of the proteasome machinery due to smoking or other factors. Persistent and elevated expression of nAChR could result in altered receptor pharmacology, increasing nicotine intake, and preventing cessation.

 In silico analysis indicated that alternative exon 5 splicing also rewires cis-motifs recognized by RNA-binding proteins (RBPs), which regulate splicing and transcript processing. Isoforms enriched by CSC exposure (FL, R372, W236) were predicted to include 10 sites for seven RBPs. These include high-confidence binding sites for A2BP1/RBFOX1, a master regulator of neuronal alternative splicing implicated in brain excitation and nicotine dependence [41, 42], FUS (a smoke-inducible RBP linked to reduced survival in non-small cell lung cancer) [43], and PUM2 (a translational repressor connected to synaptic plasticity and addiction models) [44]. We observed no differences between the predicted RBP sites in the presence of the A or G alleles of rs16969968. While *A2BP1/RBFOX1* expression was not detected in A549 and UMUC3 cells, the expression of *FUS* and *PUM2* was detectable but was not affected by CSC treatment. Thus, this mechanism might involve tissue- and condition-specific elimination of binding sites rather than altering the expression of RBPs.

In addition to smoking behavior, *CHRNA5* has been suggested to play a role in promoting direct tumorigenic phenotypes. *CHRNA5* is significantly upregulated in 17 TCGA tumor types relative to matched normal tissues [45]. Nicotine can further increase *CHRNA5* mRNA/protein expression [46, 47] and, through α5-nAChRs, drive migration/invasion in α5-expressing cells [48–51]. Elevated *CHRNA5* expression in tumors is associated with advanced disease and decreased survival in non-small cell lung cancer patients [48, 51]. *CHRNA5* knockdown inhibits migration in various model systems, while α5-nAChR–dependent intracellular phosphorylation cascades contribute to the epithelial–mesenchymal transition (EMT) and tumor progression pathways [48–50, 52–59]. Splicing shifts favoring FL isoforms, with intact phosphorylation motifs in the ICD, could enhance oncogenic signaling capacity. In this context, splicing shifts that increase the proportion of FL transcripts, which preserve the phosphorylation of serine and tyrosine residues, could potentiate pro-oncogenic signaling.

The rs16969968-A allele is strongly associated with smoking behavior and cancer risk [7–9]. However, the functional impact of this variant depends on exon 5 splicing, which determines whether the risk codon is included in *CHRNA5* transcripts and, if so, at what ratio. The fragment with rs16969968 is eliminated in all splicing forms, making the SNP and alternative splicing mutually exclusive at the transcript level but functionally convergent. Both impair receptor function via a conservative missense variant within the ICD or via loss of multiple structural/functional domains. While splicing did not appear to be affected by the rs16969968 alleles, it can mask or override the genetic association of the variant, highlighting a strong functional role for *CHRNA5* splicing in smoking-related phenotypes, even in individuals who are noncarriers of the rs16969968-A allele. This point is particularly relevant in populations of East Asian and African ancestry, where the rs16969968-A allele is uncommon (3–4%), yet chronic heavy smoking and smoking-related disease burdens in these populations are well documented. Thus, *CHRNA5* splicing may provide an ancestry-independent pathway linking smoking exposure to nicotine dependence and cancer susceptibility. Future studies should investigate whether C*HRNA5* splicing responds to cigarette smoke exposure in a dose-dependent manner, potentially clarifying how genetic and environmental factors converge to influence addiction and cancer risk. There are multiple examples of genetic variants affecting splicing, such as in *SMN2* [60], *BRCA1/BRCA2* [61], or *OAS1* [62]. However, we present an example of splicing affecting the genetic association and functional effects of a variant within an alternatively spliced region, potentially leading to variable penetrance of the risk allele.

The lung adenocarcinoma cell line A549, which is widely used in smoking-related research, was a robust model in our experiments. In this cell line, we observed the expected upregulation of known smoking-associated genes in response to CSC treatment. In the Exontrap-*CHRNA5*-exon5 minigene experiments, we observed significant splicing alterations in cells subjected to long-term continuous exposure to CSC but not in those subjected to short-term treatment (48 h). These results could be extrapolated to human smoking behavior and the damaging effects of long-term smoking exposure, but without the significant effects of transient exposure.

The strengths of this study include combined genomic analysis and experimental validation using targeted long-read cDNA sequencing to ensure accurate resolution and quantification of splicing events in large exon 5. The use of the exon 5 minigene system enabled a mechanistic study in cell lines with low/moderate endogenous *CHRNA5* expression. In addition to clarifying the mechanisms by which smoking influences CHRNA5 function, this platform represents a versatile experimental system for probing the effects of potential factors on *CHRNA5* splicing, providing new insights into receptor regulation and potential therapeutic or preventive interventions. The use of CSC, a physiologically relevant smoke surrogate containing nicotine and other constituents, provided a controlled model for smoke exposure.

Our study has several limitations. These findings are primarily based on two cancer cell lines and may not fully recapitulate tissue-specific or in vivo splicing regulation. The CSC treatment model involves smoke exposure, but differences in exposure duration, intensity, and constituents between in vitro and in vivo settings may affect its relevance. Multiple individual CSC components, or their combination, could contribute to the observed effect on *CHRNA5* splicing. Our analysis focused on exon 5 isoforms containing rs16969968, although additional *CHRNA5* splicing events may occur under certain conditions. For example, we did not analyze a recently described isoform, *CHRNA5[AluSz]*, which results from the exonization of an Alu repeat in intron 5 [63]. Similar to other alternative isoforms we explored, this common isoform results in premature termination and loss of the last TM domain, functionally affecting the resulting CHRNA5 protein.

Our AEI results were obtained in the SH-SY5Y cell line, which was selected for its high endogenous *CHRNA5* expression and the rs16969968-A/G genotype. SH-SY5Y can be induced for neuronal differentiation by 4–6 days of treatment with retinoic acid or its derivatives dissolved in DMSO. Low DMSO exposure (~ 0.1% for 4 days) did not induce neuronal differentiation [64], in contrast with high-dose DMSO treatment (1.2%, which is ~ 20-fold higher than our dose of 0.0625%) [65]. The potential effects of long-term treatment at low DMSO concentrations (> 1 month at 0.0625% in our experiments) remain unknown. Retinoic acid treatment induced a significant shift in the ratio of *CHRNA5* exon 5 splicing forms, but did not affect AEI at rs16969968 compared to control conditions. The decrease in rs16969968-A risk-allele ratio with long-term low-dose DMSO treatment in our experiments might indicate adaptation to prolonged solvent exposure, but is unrelated to smoking.

## Conclusions

Our integrative analyses revealed the diversity of *CHRNA5* exon 5 splicing, demonstrated its modulation by cigarette smoke concentrate exposure, and mapped its potential consequences for nAChR assembly, stability, and signaling. These findings indicate that smoking-induced splicing changes are a possible contributor to nicotine dependence and cancer risk, operating independently of, yet complementary to, the well-known rs16969968 variant.

## Supplementary Information

Below is the link to the electronic supplementary material.

Supplementary Material 1.

Supplementary Material 2.

## Data Availability

The data generated in this work are available from the SRA/NCBI BioProject PRJNA1322568.
