# Supplementary material for: Smoking exposure alters splicing of the nicotinic acetylcholine receptor subunit CHRNA5
Source: Hum Genomics. 2026 Jun 16;20:129. doi: 10.1186/s40246-026-01004-y (PMC13508438; doi:10.1186/s40246-026-01004-y)
Supplement: Supplementary file 1 [file 40246_2026_1004_MOESM1_ESM.docx]

**Supplementary Figures**

**Supplementary Figure S1. Validation of rs16969968 genotypes in UMUC3, A549, and SH-SY5Y cells.**

IGV plots for whole-genome sequencing in UMUC3, A549, and SH-SY5Y cell lines demonstrating genotypes of rs16969968 in *CHRNA5*-exon 5 (GRCh38; chr15:78,590,568–78,590,597). Data sources: SRA NCBI BioProject PRJNA1178369 for A549, BioProject PRJNA1134701 for UMUC3, and CCLE-public for SH-SY5Y (https://app.terra.bio/#workspaces/broad-firecloud-ccle/CCLE-public).

**Supplementary Figure S2. *CHRNA5* expression in 19,788 samples representing 54 normal human tissues from 946 individuals in GTEx.**

*CHRNA5* expression is reported as transcripts per million (TPM). Among the tissue types shown, EBV-transformed lymphocytes display the highest *CHRNA5* expression (median TPM = 3.02, n = 327). Source: Genotype-Tissue Expression (GTEx) portal, V10, https://gtexportal.org/home/gene/CHRNA5

**Supplementary Figure S3**. IGV visualization of representative RNA-seq reads corresponding to internal splicing within *CHRNA5*-exon 5 in the SH-SY5Y cell line. Alternative splicing starts at amino acids W236 or P238 and ends at G279, corresponding to out-of-frame splicing of 122 or 128 bp. Quantification of these isoforms in SH-SY5Y, A549 and UMUC3 cell lines is presented in **Table S4.**

**Supplementary Figure S4. RNA-seq analysis of endogenous *CHRNA5* splicing in A549 and UMUC3 cell lines.**

Visualization of *CHRNA5* splicing patterns using IGV Sashimi plots. A549 and UMUC3 cells were treated with cigarette smoke concentrate (CSC, 25 ng/ml) in DMSO or DMSO alone for ≥ 30 days in three biological replicates and analyzed by RNA-seq. Splicing variation in both conditions is predominantly limited to exon 5, generating the full-length and five main alternative isoforms (**Table S4**), which are also observed in TCGA tumors (Fig. 3). The isoforms are labeled based on the last amino acid retained within exon 5. The table shows average ratios across triplicates based on splice junction counts. Due to the relatively low endogenous expression of *CHRNA5*, the isoform ratios were not significantly different between the CSC/DMSO and DMSO groups.

**Supplementary Figure S5. *CHRNA5* isoform and rs16969968-A allelic ratios in SH-SY5Y cells at baseline and upon neuronal differentiation induced by retinoic acid.**

**a)** Exon junction count ratios for *CHRNA5* isoforms; **b)** rs16969968-A allele fraction in genomic DNA (WGS) and in cells treated with control or retinoic acid (RNA-seq). Individual data points represent biological replicates, with boxes indicating the interquartile range and the median (line). P-values are for two-sided Student’s t-tests. Data source: WGS: CCLE Terra, RNA-seq: SRA BioProject PRJEB44501.

**Supplementary Figure S6. Agarose gel analysis of Exontrap-*CHRNA5*-exon5-minigene RT‒PCR products.**

Agarose gels of RT‒PCR products from CSC, DMSO, empty Exontrap vector controls, and negative controls (water) from the A549 and UMUC3 cell lines. The RT‒PCR products are within the expected size range between 291 bp (empty vector amplicon), 1078 bp (reference FL transcript with spliced out introns), and 3594 bp (reference FL transcript with unspliced introns).

**Supplementary Figure S7. *CHRNA5*-exon 5 splice junction ratios in the A549 cell line subjected to short-term treatment with CSC or DMSO vehicle control.**

Results of isoform-specific TaqMan expression assays. Fold change in the isoform ratios between the CSC- and DMSO-treated A549 cells (n = 3) was quantified in the cDNA of cells transfected with Exontrap-*CHRNA5*-exon5, similar to the results shown in **Fig. 4b**. The cells were treated with CSC (25 ng/ml)/DMSO or DMSO for 48 hr. Individual data points represent biological replicates, with means (red lines) and standard deviations (error bars). P-values are for two-sided Student’s t-tests.

**Supplementary Figure S8. Splicing patterns of the Exontrap-*CHRNA5*-exon5 minigenes with rs16969968-G and A alleles** **in A549 cells.**

Isoform ratios generated by Exontrap-*CHRNA5*-exon5 minigenes with **a**) the rs16969968-A or **b**) G allele in A549 cells long-term treated with CSC and DMSO (n = 3). Splicing patterns were quantified using isoform-specific TaqMan assays and presented as fold changes relative to the corresponding DMSO-treated controls. Individual data points represent biological replicates, with means (red lines) and standard deviations (error bars). P-values are for two-sided Student’s t-tests.
